# How Do Shipworms Eat Wood? Screening Shipworm Gill Symbiont Genomes for Lignin-Modifying Enzymes

**DOI:** 10.3389/fmicb.2021.665001

**Published:** 2021-07-12

**Authors:** Stefanos Stravoravdis, J. Reuben Shipway, Barry Goodell

**Affiliations:** ^1^Goodell Laboratory, Department of Microbiology, University of Massachusetts Amherst, Amherst, MA, United States; ^2^Centre for Enzyme Innovation, School of Biological Sciences, Institute of Biological and Biomedical Sciences, University of Portsmouth, Portsmouth, United Kingdom

**Keywords:** Teredinidae, wood-borers, biodegradation, CAZymes, ligninase, laccase, peroxidase, gill endosymbionts

## Abstract

Shipworms are ecologically and economically important mollusks that feed on woody plant material (lignocellulosic biomass) in marine environments. Digestion occurs in a specialized cecum, reported to be virtually sterile and lacking resident gut microbiota. Wood-degrading CAZymes are produced both endogenously and by gill endosymbiotic bacteria, with extracellular enzymes from the latter being transported to the gut. Previous research has predominantly focused on how these animals process the cellulose component of woody plant material, neglecting the breakdown of lignin – a tough, aromatic polymer which blocks access to the holocellulose components of wood. Enzymatic or non-enzymatic modification and depolymerization of lignin has been shown to be required in other wood-degrading biological systems as a precursor to cellulose deconstruction. We investigated the genomes of five shipworm gill bacterial symbionts obtained from the Joint Genome Institute Integrated Microbial Genomes and Microbiomes Expert Review for the production of lignin-modifying enzymes, or ligninases. The genomes were searched for putative ligninases using the Joint Genome Institute’s Function Profile tool and blastp analyses. The resulting proteins were then modeled using SWISS-MODEL. Although each bacterial genome possessed at least four predicted ligninases, the percent identities and protein models were of low quality and were unreliable. Prior research demonstrates limited endogenous ability of shipworms to modify lignin at the chemical/molecular level. Similarly, our results reveal that shipworm bacterial gill-symbiont enzymes are unlikely to play a role in lignin modification during lignocellulose digestion in the shipworm gut. This suggests that our understanding of how these keystone organisms digest and process lignocellulose is incomplete, and further research into non-enzymatic and/or other unknown mechanisms for lignin modification is required.

## Introduction

Shipworms (Teredinidae) are aggressive wood-boring and wood-digesting bivalves. Their ability to rapidly degrade submerged wooden structures (such as wooden ships), coastal infrastructure (piers, jetties, and sea defenses), and aquaculture equipment has changed the course of human history and continues to cause billions of dollars in damage around the world every year (Nair and Saraswathy, 1971; Distel, 2003). Shipworms also provide invaluable ecosystem services, including the promotion of bioerosion (Davidson et al., 2018), habitat creation (Hendy et al., 2014; Shipway et al., 2019), and nutrient cycling (Cragg et al., 2020) – services which are often overlooked in light of their economic impact.

Shipworms settle on and begin to excavate into wood as larvae. After metamorphosis, the animals continue to burrow and consume wood, eventually becoming elongate and worm-like (Turner, 1966). Using highly specialized valves as drill bits, the animals produce wood shavings that are ingested, with digestion taking place in a specialized cecum (Turner, 1966; Nair and Saraswathy, 1971). Indigestible pellets of wood residue are subsequently expelled into the water column as “frass” (Turner, 1966). Over time, a colony of shipworms will rapidly degrade the inner volume of wood, leaving behind a labyrinth of excavated tunnels. Shipworms are unique because few microorganisms, or even animals, have evolved the ability to feed on the rich polysaccharide matrix locked away in woody plant biomass (Cragg et al., 2015), also known as lignocellulose. This is because lignocellulose is a complex structure, consisting of crystalline cellulose coated with both branching hemicellulose chains and encrusting, amorphous lignin; the latter being highly recalcitrant to degradation (Cragg et al., 2020; Goodell, 2020).

O’Connor et al. (2014) have documented that bacterium residing within a specialized region of the shipworm gill produce carbohydrate active enzymes (CAZymes) that are transported to the cecum for the purpose of cellulose deconstruction. These same symbionts have been thought to produce low molecular weight (LMW) metabolites that have been proposed to contribute to microbial suppression in the host cecum – the major site of wood digestion – which is nearly void of resident gut microbiota (Betcher et al., 2012; Elshahawi et al., 2013; Han et al., 2013). In a later study, Sabbadin et al. (2018) described how the shipworm hosts endogenously produce CAZymes which, supplemented by the gill bacteria CAZymes, ultimately allow for the breakdown of cellulose components of wood. This relationship between the shipworm and its gill endosymbionts lends to the favorable, efficient degradation of wood, unlocking valuable resources and energy for the host animal. However, despite the efforts of O’Connor et al. (2014) and Sabbadin et al. (2018), neither study had demonstrated a clear mechanism to remove or degrade the lignin which encases the cellulose macrofibrils.

Research on wood digestion in shipworms has primarily focused on cellulose degradation with limited consideration of lignin being a key barrier in woody biomass for most organisms. Chemical analysis of shipworm frass has been limited (Dore and Miller, 1923; Miller and Boynton, 1926; Dean, 1978; Pesante, 2018; Sabbadin et al., 2018), with cellulose and hemicellulose degradation being well known, and with more limited degradation of lignin reported. Analysis for lignin degradation can be challenging however because: (1) depolymerized lignin monomers often repolymerize during degradation to appear chemically similar to native lignin, and (2) exact amounts of wood taken in the shipworms can be difficult to estimate, and thus the amount of lignin removed in digestion is difficult to estimate. Nonetheless, understanding lignin depolymerization is critical as cellulose cannot be deconstructed until the lignin barrier is circumvented or degraded, and this is true in xylotrophic organisms ranging from termites (Geib et al., 2008; Tarmadi et al., 2017; Schalk et al., 2021) to fungi (Dashtban et al., 2010; Martínez et al., 2011; Goodell, 2020).

**FIGURE 1:**
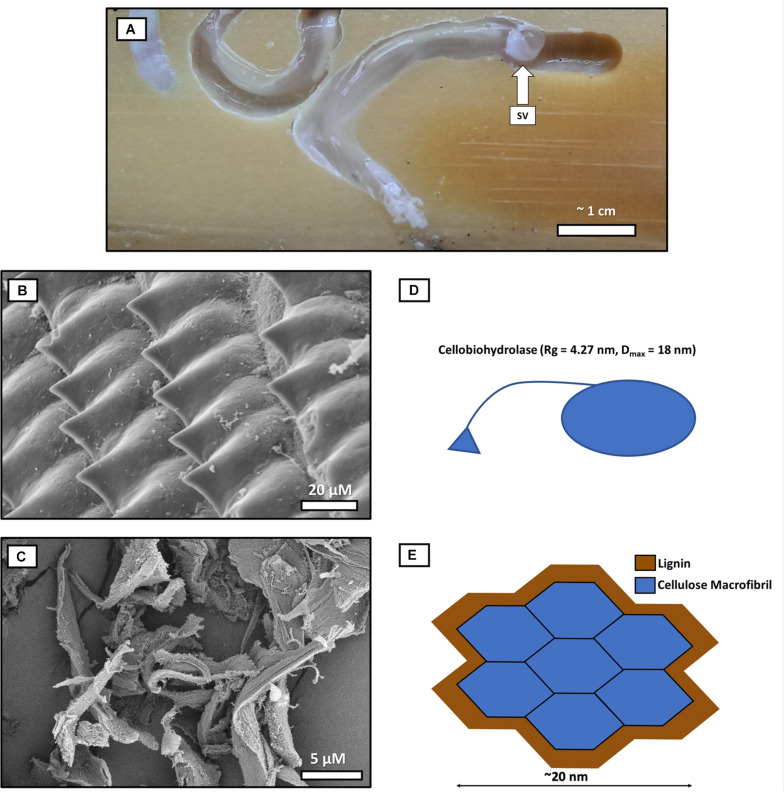
The comparative scale of a cellulose “macrofibril” and cellobiohydrolase enzyme relative to average-sized wood particles produced by shipworm shell valves. This diagram uses the 36-chain elementary fibril model for comparative purposes (Song et al., 2020) as physically, it is the largest generally accepted current model for cellulose configuration at the molecular level. **(A)** Shipworms burrowing into wood. One shipworm (right) is partially retracted in its burrow, and the boring shell valves (SV) are designated with an arrow at the anterior end of the animal. **(B)** Shipworm shell valve denticles rasp away the wood as the shipworm bores, producing **(C)** comminuted wood particles (measuring approximately 20 μm) which enter the gut. **(D)** Cellulose macrofibril (blue) encrusted with lignin (brown). Both the macrofibril and the cellobiohydrolase, **(E)** are ∼1,000 times smaller than the average wood particle produced by the rasping action of shipworm’s valves. Cellobiohydrolase and other CAZymes are unable to initiate digestion of even the relatively large cellulose macrofibril models until the encrusting lignin is removed by chemical or enzymatic action (Cellulose elementary fibril models with 18 or 24 chains would be smaller yet in comparison to even the smallest wood particles produced by shipworms in the shell grinding/comminution process). Comminution of wood by the shipworm valves does not alter the chemistry of lignin, and literature does not support that comminution would create required accessibility to cellulose at the nano-scale. SEM prep for images **B,C**: Shipworms and digestive tissue/frass was fixed in glutaraldehyde and post-fixed in osmium tetroxide before critical point drying. Imaging was conducted using a Hitachi S-4800 field emission scanning electron microscope.

Shipworms use modified shell valves to rasp wood, and some studies have suggested that this physical grinding process modifies lignin to the extent that cellulose would be accessible to enzymatic attack (Cragg et al., 2015). However, this view is not well supported at the molecular and nanoscale levels (Figure 1; Zhu et al., 2020). Further, no previous studies have assessed the mechanisms that shipworms or their symbionts use to modify and/or break down lignin. Lignin makes up approximately 30% of plant lignocellulosic materials and is composed of substituted aromatic rings with random cross-linkages, complicating its breakdown (Fuchs et al., 2011; Christopher et al., 2014; de Gonzalo et al., 2016; Lee et al., 2019; Cragg et al., 2020). The recalcitrance of lignin in microbial digestion of wood is well known (Fuchs et al., 2011; Cragg et al., 2015, 2020; de Gonzalo et al., 2016; Goodell et al., 2017; Lee et al., 2019; Goodell, 2020; Zhu et al., 2020).

Although there has been more focus in the literature on fungal deconstruction of lignin in biomass, some bacteria possess their own set of enzymes for lignin depolymerization, with eight of these being particularly noteworthy (de Gonzalo et al., 2016; Lee et al., 2019). Some of the lignin-degrading enzymes isolated from bacteria include the dye-decolorizing (DyP) peroxidases, with a heme-group that catalyzes the oxidation of lignin carbon-carbon bonds in the presence of hydrogen peroxide (Scully et al., 2013; Chen et al., 2015; Min et al., 2015; de Gonzalo et al., 2016; Janusz et al., 2017; Lee et al., 2019). Catalase-peroxidases are another group of heme-containing proteins that degrade the tough cross-linkages in lignin (Brown et al., 2011; de Gonzalo et al., 2016). Laccases represent a significant group of copper-possessing polyphenol oxidases which, in conjunction with a mediator molecule, have been produced by a wide variety of organisms to degrade even non-phenolic lignins (Sanchez-Amat and Solano, 1997; Solano et al., 2000; ten Have and Teunissen, 2001; Rosconi et al., 2005; McMahon et al., 2007; Tartar et al., 2009; Coy et al., 2010; Luna-Acosta et al., 2010; Hongoh, 2011; Scully et al., 2013; Christopher et al., 2014; Li et al., 2015; de Gonzalo et al., 2016; Lee et al., 2019; Janusz et al., 2020). Laccases have been documented in fungi and bacteria alike, including the marine bacterial genera *Alteromonas* and *Marinomonas* (Sanchez-Amat and Solano, 1997; Solano et al., 2000). Interestingly, select animals, such as certain termites and a species of sponge, have also been shown to produce laccases for wood feeding and antimicrobial activity (Geib et al., 2008; Tartar et al., 2009; Coy et al., 2010; Hongoh, 2011; Cragg et al., 2015; Li et al., 2015; Besser et al., 2018; Janusz et al., 2020). A fourth group of proteins associated with lignin degradation are manganese cofactor-dependent superoxide dismutases, a class of proteins normally used to convert ROS into less harmful forms in order to reduce cellular stress (Rashid et al., 2015; de Gonzalo et al., 2016; Lee et al., 2019). In some bacteria, these proteins can be secreted from the cell to interact with dioxygen in the presence of wood to produce a reactive species and break lignin bonds (Rashid et al., 2015). Various glutathione-dependent β-etherases (denoted as LigE, LigF, and LigP) have also been shown to modify lignin and its partially degraded intermediates by cleaving the abundant β-*O*-4 aryl ether bonds present throughout lignin’s complex structure (Allocati et al., 2009; Scully et al., 2013; Picart et al., 2015; de Gonzalo et al., 2016; Lee et al., 2019; Voß et al., 2020). Classes of dioxygenases (intradiol, extradiol, and LigAB) have also been documented to attack a variety of aromatic substances, including lignin and its derivatives (Sugimoto et al., 1999; Sonoki et al., 2009; Barry and Taylor, 2013; Bianchetti et al., 2013; Scully et al., 2013; de Gonzalo et al., 2016; Burroughs et al., 2019; Lee et al., 2019). As demonstrated by Bianchetti et al. (2013), a protein possessing just an extradiol dioxygenase domain and a carbohydrate binding site can target and begin breaking down lignocellulose. Lastly, the heme-possessing lignin peroxidases and manganese peroxidases (ten Have and Teunissen, 2001; Lambertz et al., 2016; Kumar and Chandra, 2020) have been found in select bacteria, such as species of *Streptomyces* (Jing, 2010; Jing and Wang, 2012), *Comamonas* (Chen et al., 2012), and termite endosymbionts (Zhou et al., 2017). These two enzymes have been largely documented in fungi (Harris et al., 1991; ten Have and Teunissen, 2001; de Gonzalo et al., 2016; Lambertz et al., 2016; Lee et al., 2019; Kumar and Chandra, 2020), where lignin peroxidases have strong activity against lignin and drive oxidation of phenolic and non-phenolic bonds using hydrogen peroxide and a mediator (ten Have and Teunissen, 2001; Jing, 2010; Jing and Wang, 2012; de Gonzalo et al., 2016; Kumar and Chandra, 2020). Manganese peroxidases similarly target phenolic and non-phenolic bonds present throughout lignin by initially oxidizing a manganese ion, which then attacks the lignin and oxidizes the tough polymer (Harris et al., 1991; ten Have and Teunissen, 2001; de Gonzalo et al., 2016; Kumar and Chandra, 2020).

Apart from the above enzymatic approaches to lignin modification, alternative mechanisms for lignin deconstruction have been demonstrated in other organisms. Wood digesting organisms must possess a system to strip away or depolymerize the encrusting lignin matrix at the molecular level to allow cellulose to be accessed by carbohydrate active enzymes (CAZymes). For example, limnorids, a group of marine crustaceans, have been shown to secrete hemocyanin into their gut, making lignin more porous and allowing cellulases to move in and bind the cellulose components of the wood cell wall (Besser et al., 2018).

In fungi, diverse systems are available for tackling the lignin problem. For instance, white rot fungi are known to use a variety of extracellular enzymes to deconstruct this lignin barrier, such as lignin peroxidases, manganese peroxidases, and laccases (Harris et al., 1991; ten Have and Teunissen, 2001; Eastwood, 2014; de Gonzalo et al., 2016; Lambertz et al., 2016; Lee et al., 2019; Goodell, 2020; Kumar and Chandra, 2020). These proteins, in some cases with the aid of a mediator molecule, drive oxidation of phenolic and non-phenolic lignin bonds. In concert with the lignin-degrading enzymes, an array of CAZymes, such as glycoside hydrolases and carbohydrate esterases, facilitate the depolymerization of cellulose into simpler sugars (Eastwood, 2014). Brown rot fungi, however, produce no lignin-degrading peroxidases, and they have a vastly reduced suite of glycoside hydrolases (Yelle et al., 2011; Arantes et al., 2012; Eastwood, 2014; Cragg et al., 2015, 2020; Goodell et al., 2017; Zhu et al., 2017). The brown rot fungi are perhaps the most successful destructive organisms of wood on earth, and they dominate other fungi in their aggressive attack – particularly of softwood species (Eastwood, 2014; Goodell, 2020; Umezawa et al., 2020). These fungi supplement their more limited suite of CAZymes to deconstruct lignin with a powerful non-enzymatic system, known as the chelator-mediated Fenton (CMF) system, that generates reactive oxygen species (ROS) to depolymerize both lignin and holocellulose (Goodell et al., 2017; Kirker, 2018; Goodell, 2020; Hess et al., 2020; Zhu et al., 2020). This process leads to the repolymerization of the lignin in such a way as to relocate and nodularize the modified lignin. Following expression of these CMF metabolites, brown rot fungi then express a narrowed range of CAZymes to further deconstruct the oligosaccharide residues from cellulose (Umezawa et al., 2020; Zhu et al., 2020). This coupled system of extensive, non-enzymatic deconstruction/modification of lignin in parallel with both non-enzymatic and enzymatic digestion of holocellulose is highly optimized and contributes significantly to the degradation of coniferous wood by brown rot fungi (Yelle et al., 2011; Arantes et al., 2012; Eastwood, 2014; Goodell et al., 2017; Goodell, 2020; Zhu et al., 2020). Brown rot fungi, as outlined above, produce ROS non-enzymatically to modify and relocate repolymerized, and modified lignin (Yelle et al., 2011; Arantes et al., 2012; Eastwood, 2014; Cragg et al., 2015, 2020; Goodell et al., 2017; Zhu et al., 2017, 2020).

To date, no research has assessed whether mollusk gill symbionts produce lignin-degrading enzymes even among mollusks that produce laccases (Geib et al., 2008; Coy et al., 2010; Luna-Acosta et al., 2010; Fuchs et al., 2011; Scully et al., 2013; O’Connor et al., 2014; Brito et al., 2018; Sabbadin et al., 2018; Janusz et al., 2020). Further, no research has been conducted to assess if enzymatic vs. non-enzymatic mechanisms, such as the non-enzymatic mechanisms discovered in limnorids or fungi, may be active. In addition, no research has explored whether the bacterial symbionts in shipworms participate in any type of enzymatic or non-enzymatic attack on lignin. Our study focuses on the genomes of *all* currently characterized *wood-boring* shipworm symbionts (Yang et al., 2009; O’Connor et al., 2014), including four recently described species (Altamia et al., 2020, 2021), with updated/revised sequenced genomes. This allows, for the first time, an extensive exploration for putative lignin-degrading enzymes encoded within these genomes in order to examine the role (or lack thereof) of these bacteria in removing lignin and opening up the wood for further digestion.

## Materials and Methods

### Genomic Data

Five shipworm gill symbiont genomes (Table 1) were accessed and analyzed using the Joint Genome Institute (JGI) Integrated Microbial Genomes and Microbiomes Expert Review (IMG/MER) (Chen et al., 2020; Mukherjee et al., 2020): *Teredinibacter turnerae* (Yang et al., 2009), *T. waterburyi* (O’Connor et al., 2014; Altamia et al., 2020), *T. haidensis*, *T. purpureus*, and *T. franksiae* (O’Connor et al., 2014; Altamia et al., 2021). Each genome was organized under a single Genome Set for subsequent analysis in IMG.

**TABLE 1:** Summary of shipworm symbiont bacterial genomes acquired from JGI IMG.

Genome	Shipworm host	IMG Genome ID	GOLD analysis Project I.D.	Protein coding genes	Proteins with predicted function
*Teredinibacter turnerae* T7901^1^	*Lyrodus pedicellatus*	644736410	Ga0031157	4254 (98.75%)	3083 (71.56%)
*Teredinibacter waterburyi* BS02^2^	*Bankia setacea*	2503982003	Ga0010298	3277 (98.70%)	2584 (77.83%)
*Teredinibacter haidensis* BS08^2^	*Bankia setacea*	2767802764	Ga0248310	4033 (98.80%)	2984 (73.10%)
*Teredinibacter purpureus* BS12^2^	*Bankia setacea*	2170459028	Ga0003581	4560 (98.59%)	2980 (64.43%)
*Teredinibacter franksiae* BSc2^2^	*Bankia setacea*	2531839719	Ga0015035	4837 (98.68%)	3320 (70.65%)

*^1^The *Teredinibacter turnerae* genome was generated by Yang et al. (2009).*

*^2^The genomic data for the above four strains of bacterial symbionts were found using O’Connor et al. (2014) and given species designations according to Altamia et al. (2020) and Altamia et al. (2021).*

### Functional ID Search of JGI Genomes

Every available functional domain associated with bacterial lignin depolymerization enzymes (DyP peroxidases, laccases, catalase-peroxidases, manganese-dependent superoxide dismutases, β-etherases, aromatic ring cleaving dioxygenases, lignin peroxidases, and manganese peroxidases) were found by searching through four protein function databases (Pfam, TIGRfam, COG, and KEGG ontology) within JGI IMG/MER (de Gonzalo et al., 2016; Lee et al., 2019; Chen et al., 2020; Mukherjee et al., 2020). From these four databases, terms specifically associated with the eight proteins of interest were found and organized under a single Function Set for downstream use.

Since β-etherases are part of the glutathione S-transferase (GST) family, a large protein family associated with a wide breath of functions, we could only find general functional IDs relating to GSTs (Allocati et al., 2009; Voß et al., 2020). We were also unable to find functional terms relating to lignin peroxidases or manganese peroxidases. Since there were no functional terms specific to β-etherases, lignin peroxidases, or manganese peroxidases, we did not include these proteins in our Function Profile search. The five bacterial symbiont genomes were inspected using JGI’s Function Profile tool after inputting the following: three specific functional terms for DyP-type peroxidases (*pfam04261*, *TIGR01413*, and *KO:K15733*), five specific IDs for laccases (*pfam02578*, *TIGR03389*, *COG1496*, *KO:K05810*, and *KO:K05909*), three functional terms corresponding to catalase-peroxidases (*TIGR00198*, *COG0376*, and *KO:K03782*), three terms associated with manganese-dependent superoxide dismutases (*pfam00081*, *pfam02777*, and *KO:K04564*), and 14 specific functional terms corresponding to class II and class III aromatic ring cleaving dioxygenases (*pfam07746*, *pfam02900*, *TIGR02422*, *TIGR02423*, *TIGR02792*, *COG3384*, *COG3485*, *COG3805*, *COG3885*, *KO:K00448*, *KO:K00449*, *KO:04100*, *KO:K04101*, and *KO:11945*). The latter group had the most diverse set of functional terms that could be used for our analyses.

### Blastp Analysis of JGI Genomes and Protein Modeling

Five reference sequences were found for each of the eight groups of putative lignin-degrading proteins. Protein sequences for DyP peroxidases (GenBank Accessions: WP_045819334.1, WP_015046092.1, WP_170150844.1, WP_136783674.1, and WP_061094052.1), laccases (GenBank Accessions: WP_011466803.1, PUA27947.1, WP_045826889.1, OES40331.1, and WP_018982942.1), and catalase-peroxidases (GenBank Accessions: ABD83143.1, QEI20369.1, QEI17940.1, VTP57286.1, and WP_018982669.1) were found for organisms within the same family or order as our isolate genomes (Spring et al., 2015). For the manganese-dependent superoxide dismutases, two protein sequences of *Sphingobacterium* sp. T2 were acquired from Rashid et al. (2015) (GenBank Genome Accession: JXAC00000000; GenBank Protein Accession: WP_039053709.1 and WP_039053587.1). The proteins were then blasted in the National Center for Biotechnology Information (NCBI) databases against *Gammaproteobacteria* (Taxid: 1236). A sequence with the highest percent identity match (64.32% identity; GenBank Accession: PZR21549.1) was one belonging to a member of the Enterobacteriaceae family, and this sequence as well as one from a member of the Alteromonadaceae family (61.62% identity; GenBank Accession: MAO07630.1) and one from a member of the Moraxellaceae family (60.48% identity; GenBank Accession: RZA00329.1) were used for subsequent analyses. LigE (GenBank Accession: WP_044331491.1) and LigF (GenBank Accession: OGT78215.1) β-etherase sequences from a *Sphingomonas* species and a Gammaproteobacterium were, respectively, acquired from Voß et al. (2020). A LigP (GenBank Accession: BAK67935.1) sequence and two additional β-etherases from species of *Acinetobacter* (GenBank Accession: ATY43794.1 and VAX44176.1) were obtained from the NCBI protein database. Sequences for an intradiol (GenBank Accession: WP_014046731.1) and an extradiol (GenBank Accession: WP_014046532.1) aromatic ring cleaving dioxygenase were obtained from a published genome of *Streptomyces* (GenBank Genome Accession: CP002993) generated by Bianchetti et al. (2013). The reference sequences for LigAB dioxygenases were obtained by searching against *Gammaproteobacteria* in NCBI. Specifically, the amino acid sequences for one LigA subunit (GenBank Accession: VFT07265.1) and two LigB subunits (GenBank Accessions: EJN21703.1 and EJM47535.1) from *Pseudomonas* species were obtained. Five bacterial lignin peroxidases were obtained from the NCBI protein database (GenBank Accessions: AQA21607.1, AQA24509.1, GGI48058.1, OOK77773.1, and SHD68351.1). Considering that lignin peroxidases are commonly cited in fungi (de Gonzalo et al., 2016; Lee et al., 2019), we obtained five additional protein sequences for five fungal lignin peroxidases (GenBank Accession: AAA33737.1, AAA33739.1, AAA34049.1, AAW71986.1, and CAA53333.1). Since no bacterial manganese peroxidases could be definitively identified on NCBI, we obtained the protein sequences of five fungal manganese peroxidases to be used for downstream analyses (GenBank Accessions: AAA33743.1, AAA62243.1, AAD02880.1, CAA83148.1, and XP_007370520.1).

A multi-blastp analysis was run using the default parameters on JGI IMG/MER against each of the five genomes (Chen et al., 2020; Mukherjee et al., 2020). The resulting blastp hits for all eight groups of putative ligninase-encoding genes were compared against the results of the Function Profile analysis. Using SWISS-MODEL, each protein sequence obtained from previous steps was compared to, and modeled against, reference protein templates (Guex et al., 2009; Bienert et al., 2017; Waterhouse et al., 2018; Studer et al., 2020).

## Results

### Function Profile Analysis of Lignin-Degrading Proteins

Function Profile searches showed that each of the five endosymbiont genomes possessed a limited number of genes that putatively encoded for ligninases, though most matches had low identity and poor modeling hits, as discussed further in subsequent sections of the Results and Discussion (Table 2; Chen et al., 2020; Mukherjee et al., 2020). None of the genomes had a protein sequence with DyP-type peroxidase domains, but each genome did possess a potential laccase-encoding gene and a probable manganese-dependent superoxide dismutase gene. Each symbiont had at least one gene potentially encoding a catalase-peroxidase, though the genomes of the isolates *T*. *turnerae* T7901 and *T. franksiae* BSc2 had two possible catalase-peroxidase genes. Only the genome of *T*. *turnerae* T7901 possessed genes encoding predicted ring-cleaving dioxygenases, specifically a potential extradiol dioxygenase (gene ID: 644918224) and a protein with the catalytic domain of a LigAB dioxygenase (gene ID: 644916349).

**TABLE 2:** Symbiont genes possessing protein domains/regions matching functional IDs for putative bacterial ligninases from four protein family databases.

Gene ID	Gene (IMG Product) name	Protein family (Pfam and TIGRfam) IDs	KEGG ID	Clusters of Orthologous Groups (COG) ID	Predicted protein
**Teredinibacter turnerae** **T7901**					
644916161	hypothetical protein	pfam02578	KO:K05810	COG1496	Laccase
644916349	aromatic ring-opening dioxygenase, catalytic LigB subunit family protein	pfam02900	KO:K04101	COG3384	Dioxygenase^1^
644916432	catalase/peroxidase HPI	pfam00141, TIGR00198	KO:K03782	COG0376	Catalase-Peroxidase
644917076	catalase/peroxidase HPI	pfam00141, TIGR00198	KO:K03782	COG0376	Catalase-Peroxidase
644918224	4,5-DOPA extradiol dioxygenase-like protein	pfam02900	NA	COG3384	Dioxygenase
644917936	superoxide dismutase	pfam00081, pfam02777	KO:K04564	COG0605	Superoxide Dismutase^2^
**Teredinibacter waterburyi** **BS02**					
2503998252	catalase-peroxidase	pfam00141, TIGR00198	KO:K03782	COG0376	Catalase-Peroxidase
2503998928	hypothetical protein	pfam02578	KO:K05810	COG1496	Laccase
2503998518	Fe-Mn family superoxide dismutase	pfam00081, pfam02777	KO:K04564	COG0605	Superoxide Dismutase
**Teredinibacter haidensis** **BS08**					
2770887430	catalase-peroxidase	pfam00141, TIGR00198	KO:K03782	COG0376	Catalase-Peroxidase
2770887603	hypothetical protein	pfam02578	KO:K05810	COG1496	Laccase
2770886725	Fe-Mn family superoxide dismutase	pfam00081, pfam02777	KO:K04564	COG0605	Superoxide Dismutase
**Teredinibacter purpureus** **BS12**					
2545659092	catalase-peroxidase	pfam00141, TIGR00198	KO:K03782	COG0376	Catalase-Peroxidase
2545660462	conserved hypothetical protein	pfam02578	KO:K05810	COG1496	Laccase
2545658554	superoxide dismutase, Fe-Mn family	pfam00081, pfam02777	KO:K04564	COG0605	Superoxide Dismutase
**Teredinibacter franksiae** **BSc2**					
2534573301	uncharacterized protein, YfiH family	pfam02578	KO:K05810	COG1496	Laccase
2534574671	Catalase (peroxidase I)	pfam00141	KO:K03782	NA	Catalase-Peroxidase
2534574672	catalase/peroxidase HPI	pfam00141, TIGR00198	KO:K03782	COG0376	Catalase-Peroxidase
2534576050	Superoxide dismutase	pfam00081, pfam02777	KO:K04564	COG0605	Superoxide Dismutase

*The data below was obtained using the Function Profile tool (Chen et al., 2020; Mukherjee et al., 2020) available from JGI IMG/MER.*

*^1^The functional IDs for the aromatic ring cleaving dioxygenases were specific to class III, with the exception of a COG and a KEGG term which were more general or specific to class II dioxygenases, respectively.*

*^2^Note that all superoxide dismutases of interest are manganese-dependent or associated.*

### Blastp Hits

Less than one percent of each of the five genomes (average 0.18%), were predicted to be associated with lignin degradation or modification (Supplementary Tables 1, Table 2). The same genes from the functional ID analysis (Table 2) were observed from our blastp analyses (Supplementary Tables 3–7). According to literature estimates, the average number of verified CAZymes present across these five genomes (6.31%) was noticeably higher than that of the putative ligninases (Yang et al., 2009; O’Connor et al., 2014). Each predicted ligninase significantly matched with our reference sequences (*e*-value < 0.001), though only the laccases, catalase-peroxidases, and superoxide dismutases had consistently strong alignments according to their bit scores (values > 100 and as high as ∼1100). A second possible laccase from *T*. *waterburyi* BS02 (gene ID: 2503998386) was found after blasting its genome against the reference sequence WP_011466803.1, but this match had a low percent identity of 28% (Supplementary Table 3). Only the catalase-peroxidases had average percent identities greater than 50% (Supplementary Table 4). We blasted our genomes against five β-etherase reference sequences derived from Voß et al. (2020) and NCBI GenBank. The LigF β-etherase sequence (OGT78215.1) produced blastp hits against at least one gene in each genome, with the exception of *T*. *waterburyi* BS02 (Supplementary Table 6). The *T*. *haidensis* BS08 isolate had two potential LigF β-etherases while the *T. purpureus* BS12 genome had four probable β-etherases; however, all of these genes had percent identities of less than 30%. There was at least one gene from each endosymbiont that blasted against the bacterial lignin peroxidases (two from *T*. *waterburyi* and *T*. *haidensis*, four from *T*. *turnerae*), though each gene had low percent identities of less than 40% (Supplementary Table 8). Each gene was identified as either an adenylate cyclase, a tetratricopeptide repeat domain protein, or an alpha/beta hydrolase family protein. There were no hits from our fungal blastp check step, and there were no manganese peroxidases predicted from our blastp analysis.

### Protein Modeling Hits

None of the putative laccases, catalase-peroxidases, superoxide dismutases, and β-etherases matched to reference protein templates using SWISS-MODEL (Guex et al., 2009; Bienert et al., 2017; Waterhouse et al., 2018; Studer et al., 2020). The two putative dioxygenases from *T*. *turnerae* T7901 (gene IDs: 644918224 and 644916349) matched to a protein template of an iron-free 1,6-APD 2-aminophenol-1,6-dioxygenase (Li et al., 2013). Only the predicted extradiol dioxygenase (gene ID: 644918224) could be modeled using the protein template. However, the sequence identities for both proteins were low (17.65 and 16.53%, respectively) and the protein model was low quality (QMEAN < −4.0), indicating unreliability in the predictions (Guex et al., 2009; Bienert et al., 2017; Waterhouse et al., 2018; Studer et al., 2020). In *T*. *turnerae* and *T*. *waterburyi*, none of the putative lignin peroxidases could be matched to reference templates. The two potential lignin peroxidases from *T*. *haidensis* matched with over 50 protein templates and two to three protein models for adenylate cyclases (sequence identities between 23 and 67%; QMEAN for all >−4.0) (Topal et al., 2012; Barathy et al., 2014, 2015; Etzl et al., 2018). Similarly, the potential lignin peroxidases from *T*. *purpureus* and *T*. *franksiae* both matched to over 50 protein templates. The former was modeled after two adenylate cyclases (sequence identities < 28%; QMEAN for all > −4.0) (Barathy et al., 2014, 2015) while the latter was modeled from three adenylate cyclases (sequence identities < 28%; QMEAN < −4.0 for two of the models) (Barathy et al., 2014; Lindner et al., 2017). To our knowledge, adenylate cyclases have no involvement in lignin modification.

## Discussion

In bacteria, eight major protein families have been identified to date as being responsible for lignin degradation or modification (de Gonzalo et al., 2016; Lee et al., 2019). Following our functional ID and blastp analyses, we identified that each shipworm bacterial symbiont possessed at least four genes encoding a predicted putative ligninase (Supplementary Tables 1, 2). The blastp analyses using NCBI reference sequences uncovered an additional putative laccase in the *T*. *waterburyi* isolate which was not detected by our Function Profile search (Supplementary Table 3; Chen et al., 2020; Mukherjee et al., 2020). Similarly, at least one predicted β-etherase and one potential lignin peroxidase were found in most isolate genomes using the blastp analysis (Supplementary Tables 6, 8). However, there is low confidence in these results, as our proteins had generally low percent identities aduce high quality protein models via SWISfter each blastp search and we were largely unable to proS-MODEL (Supplementary Tables 3, 8; Guex et al., 2009; Bienert et al., 2017; Waterhouse et al., 2018; Studer et al., 2020). In the case of our potential lignin peroxidases, none of our protein models matched known ligninases, lowering confidence in their involvement in lignin modification. If expressed, the recorded enzymes could potentially be involved in lignin degradation, but these proteins cannot be clearly linked to this function without experimental verification. Though putatively possessing the correct domains, these proteins may simply fulfill other cellular activities, such as reducing oxidative stress in the gill environment (Rashid et al., 2015; de Gonzalo et al., 2016; Lee et al., 2019). Previous studies support this concept. For instance, bacterial laccases operate intracellularly and in neutral to basic environments (Rosconi et al., 2005; McMahon et al., 2007; Christopher et al., 2014). As such, the secretion of this protein and its transport to the host shipworm’s acidic digestive system would present numerous challenges (Nair and Saraswathy, 1971).

The ability of shipworms to consume wood is a complicated process which, to circumvent the lignin barrier to access cellulose, requires involvement of one or more mechanisms for lignin modification or degradation. It is important to note that mechanical comminution of wood alone has been well established in other related systems to be insufficient in facilitating enzymatic access to cellulose without pre-processing lignin (Tepfer and Taylor, 1981; Flournoy et al., 1991; Kleman-Leyer et al., 1992; Arantes et al., 2012; Cragg et al., 2015; Goodell et al., 2017; Zhu et al., 2020). Unless lignin has been first deconstructed or digested, cellulose-degrading enzymes are unable to successfully pass through into unmodified plant cell walls of any type, and research demonstrates that unmodified cell walls are not degraded by CAZymes alone (Flournoy et al., 1991; Zhu et al., 2020).

In general, wood-feeding animals approach lignin digestion through the use of gut-residing, symbiotic microbes, which secrete extracellular enzymes into the gut to modify the lignin (Geib et al., 2008; Coy et al., 2010; Fuchs et al., 2011; Scully et al., 2013; Cragg et al., 2015). Additionally, animals have been shown to use other tactics for degrading lignin (Schalk et al., 2021). Similar to shipworms, some insects physically chew and grind the wood into smaller particles to help open the structure for lignin- and cellulose-modification (Cragg et al., 2015), but they must have enzymatic or non-enzymatic systems to further deconstruct lignin at the molecular level. In termites, this mechanism is coupled with CAZyme and endogenous ligninase production; the latter of which is not present in the shipworm gut (Geib et al., 2008; Tartar et al., 2009; Coy et al., 2010; Hongoh, 2011; Cragg et al., 2015). In limnorid crustaceans, a non-enzymatic system involving hemocyanin also has been reported for degradation of lignin (Besser et al., 2018). Lignin is exposed to the hemocyanin and becomes increasingly porous, allowing cellulases and other essential proteins to access the remaining cell wall sugars (Besser et al., 2018). In some sense, the hemocyanin mechanism in limnorids is a similar non-enzymatic strategy to the CMF mechanism employed by brown rot fungi to circumvent lignin which encrusts cellulose (Yelle et al., 2011; Arantes et al., 2012; Eastwood, 2014; Cragg et al., 2015, 2020; Goodell et al., 2017; Zhu et al., 2017; Besser et al., 2018; Goodell, 2020).

The results outlined herein suggest that shipworm bacterial gill symbionts do not produce lignin-degrading extracellular enzymes. Similarly, previous transcriptomic analyses (Honein et al., 2012; Sabbadin et al., 2018) have also similarly failed to identify endogenously produced host ligninases. Despite these results, shipworms are clearly able to deconstruct lignin and access cellulose. As such, we suggest that an alternative, enzymatic or non-enzymatic system, perhaps similar to those employed by select fungi, must exist (Yelle et al., 2011; Arantes et al., 2012; Eastwood, 2014; Cragg et al., 2015, 2020; Goodell et al., 2017; Zhu et al., 2017, 2020; Goodell, 2020; Hess et al., 2020).

As the primary degraders of lignocellulose in aquatic environments, shipworms play a fundamental role in cycling woody plant material across a range of ecosystems, including terrestrial wood deposits in shallow coastal seas, mangrove forests, and seagrass rhizomes (Hendy et al., 2014; Shipway et al., 2016; Davidson et al., 2018; Cragg et al., 2020). Yet, our understanding of wood digestion by shipworms and other organisms in marine ecosystems is incomplete and has been comparatively neglected considering the extensive research into terrestrial wood degradation. Research in this area should continue to explore the mechanisms of wood digestion in shipworms, with particular focus on lignin degradation. We also note that this research bears relevance to industry including affordable, energy-efficient removal and processing of lignin which could aid the production of biofuels and other valuable products (Arantes et al., 2012; Christopher et al., 2014; Eastwood, 2014; Picart et al., 2015; de Gonzalo et al., 2016; Lee et al., 2019; Zhu et al., 2020). Finally, examination of lignin digestion in modified timbers, such as acetylation (Pawar et al., 2013; Klüppel et al., 2015) or furfurylation (Slevin et al., 2015; Thygesen et al., 2020), may prove useful in developing our understanding of lignin processing in shipworms, and lead to the development of wood products resistant to shipworm attack.

## Data Availability Statement

The original contributions presented in the study are included in the article/Supplementary Material, further inquiries can be directed to the corresponding author/s.

## Author Contributions

JS and BG conceptualized this project. SS analyzed the data and wrote the manuscript, with significant contributions, editing, and insight provided by each co-author. All authors contributed to the article and approved the submitted version.

## Conflict of Interest

The authors declare that the research was conducted in the absence of any commercial or financial relationships that could be construed as a potential conflict of interest.
